# Blue light flexible cystoscopy with hexaminolevulinate in non-muscle-invasive bladder cancer: review of the clinical evidence and consensus statement on optimal use in the USA — update 2018

**DOI:** 10.1038/s41585-019-0184-4

**Published:** 2019-04-24

**Authors:** Yair Lotan, Trinity J. Bivalacqua, Tracy Downs, William Huang, Jeffrey Jones, Ashish M. Kamat, Badrinath Konety, Per-Uno Malmström, James McKiernan, Michael O’Donnell, Sanjay Patel, Kamal Pohar, Matthew Resnick, Alexander Sankin, Angela Smith, Gary Steinberg, Edouard Trabulsi, Michael Woods, Siamak Daneshmand

**Affiliations:** 1 UT Southwestern Medical Center, Dallas, TX USA; 2 James Buchanan Brady Urological Institute, Johns Hopkins University, Baltimore, MD USA; 3 Department of Urology, University of Wisconsin, Madison, WI USA; 4 Department of Urology, New York University School of Medicine, New York, NY USA; 5 Genitourinary Surgery Section, Michael E. DeBakey VA Medical Center, Houston, TX USA; 6 Department of Urology, University of Texas MD Anderson Cancer Center, Houston, TX USA; 7 Department of Urology, University of Minnesota, Minneapolis, MN USA; 8 Department of Urology, Institute of Surgical Sciences, Uppsala University, Uppsala, Sweden; 9 Department of Urology, Columbia University Medical Center, New York City, NY USA; 10 Department of Urology, University of Iowa, Iowa City, IA USA; 11 Department of Urology, University of Oklahoma, Oklahoma City, OK USA; 12 Department of Urology, Ohio State University, Columbus, OH USA; 13 Department of Urologic Surgery, Vanderbilt University, Nashville, TX USA; 14 Department of Urology, Montefiore Medical Center, New York, NY USA; 15 Department of Urology, University of North Carolina, Chapel Hill, NC USA; 16 Department of Urology, University of Chicago, Chicago, IL USA; 17 Department of Urology, Sidney Kimmel Medical College at Thomas Jefferson University, Philadelphia, PA USA; 18 Department of Urology, Loyola University Chicago, Chicago, IL USA; 19 Department of Urology, University of Southern California, Los Angeles, CA USA

**Keywords:** Bladder cancer, Cancer imaging, Endoscopy

## Abstract

Blue light cystoscopy (BLC) with hexaminolevulinate (HAL) during transurethral resection of bladder cancer improves detection of non-muscle-invasive bladder cancer (NMIBC) and reduces recurrence rates. Flexible BLC was approved by the FDA in 2018 for use in the surveillance setting and was demonstrated to improve detection. Results of a phase III prospective multicentre study of blue light flexible cystoscopy (BLFC) in surveillance of intermediate-risk and high-risk NMIBC showed that 20.6% of malignancies were identified only by BLFC. Improved detection rates in the surveillance setting are anticipated to lead to improved clinical outcomes by reducing future recurrences and earlier identification of tumours that are unresponsive to therapy. Thus, BLFC has a role in surveillance cystoscopy, and determining which patients will benefit from BLFC and optimal and cost-effective ways of incorporating this technology into surveillance cystoscopy must be developed.

## Abstract (web summary)

In this Consensus Statement, experts in the field detail the current evidence regarding blue light flexible cystoscopy for bladder cancer surveillance and make recommendations regarding its use on the basis of conclusions arrived at during the panel discussions at a consensus meeting.

## Introduction

Approximately 75% of newly diagnosed bladder cancers are not muscle invasive at presentation^1^. Non-muscle-invasive bladder cancer (NMIBC) has a high recurrence rate (50–70% of patients), and 10–20% of NMIBCs (especially high-risk disease) will progress to muscle-invasive disease (depending on stage and grade at diagnosis)^1^. Initial management of NMIBC includes transurethral resection of bladder tumour (TURBT). Use of intravesical BCG or intravesical chemotherapy in intermediate-risk and high-risk disease decreases the risk of recurrence and progression^2,3^. Surveillance protocols for patients with NMIBC involve frequent cystoscopic evaluation primarily in the outpatient setting^2,4^. White light cystoscopy (WLC) has long been the standard-of-care modality for surveillance with a high sensitivity for detection of papillary tumours^2,4^. A known limitation of WLC is in detecting carcinoma in situ (CIS), which can result in a false-negative rate as high as 20%^5,6^. Thus, some patients with recurrence are missed and might progress to worse disease as their disease was unrecognized.

The inadequacy of WLC to visualize tumours has led to the development of enhanced cystoscopic techniques. The goal of these techniques is to reduce early recurrences, as many are tumours that were missed or inadequately initially resected owing to suboptimal visualization^7^. WLC remains the standard-of-care technique for the detection of bladder cancer, but extensive data have shown that enhanced cystoscopy with blue light frequently detects tumours that are missed by white light^8^. The American Urological Association (AUA)–Society of Urologic Oncology (SUO) guidelines for managing NMIBC state that “in a patient with NMIBC, a clinician should offer blue light cystoscopy at the time of TURBT, if available, to increase detection and decrease recurrence. (Moderate Recommendation; Evidence Strength: Grade B)”^2^. The European Association of Urology (EAU) guidelines also state that fluorescence-guided biopsy and resection are more sensitive than conventional procedures for the detection of malignant tumours, particularly for CIS (evidence level: 1a)^4^.

Outpatient white light flexible cystoscopy (WLFC) is the gold-standard procedure for surveillance of patients with NMIBC^9^. Given the likelihood of missed tumours in the crucial surveillance setting and the evidence of improved detection with blue light cystoscopy (BLC) in the operative setting, blue light flexible cystoscopy (BLFC) for surveillance in the outpatient setting clearly has potential to improve detection of recurrent tumours. A 2018 prospective phase III clinical study in the USA evaluated the use of BLFC for patients with intermediate-risk or high-risk NMIBC in surveillance in the outpatient setting and found that 20.6% of malignancies were identified only by BLFC^10^. A consensus group met at AUA 2018 in San Francisco, USA, following publication of the trial to review the growing body of data for BLC and BLFC internationally and consider its utility in various clinical areas. This Consensus Statement details the current evidence regarding BLFC for bladder cancer surveillance and makes recommendations regarding use on the basis of conclusions arrived at during the panel discussions.

## Methods

For purposes of this Consensus Statement, a meeting was held at the AUA Meeting on 17 May 2018. The meeting consisted of 17 specialists in bladder cancer who are experienced with the use of BLC, including 14 who participated in the phase III BLFC for surveillance trial^10^. Before the meeting, a survey of the panel was performed regarding which patients the panel anticipated would be suitable to undergo BLFC for surveillance, the time intervals after TURBT and during surveillance at which the procedure would be performed and the special clinical scenarios, such as positive cytology and office fulguration or biopsy. Questions about logistical and financial concerns were also posed.

The consensus meeting included extensive discussion about the current state of the literature and reviewed the highest level of evidence involving BLC for TURBT and BLFC for surveillance in the outpatient setting as well as future directions. The survey results were discussed and a consensus was developed regarding optimal use of BLFC for surveillance on the basis of current knowledge. During drafting of this Consensus Statement, the authors performed a PubMed search using the terms “bladder cancer”, “hexaminolevulinate blue light cystoscopy”, “hexvix”, “cysview” and “photodynamic diagnosis” and reviewed currently available guidelines on NMIBC.

## Background

### Blue light cystoscopy

**Fig. 1:**
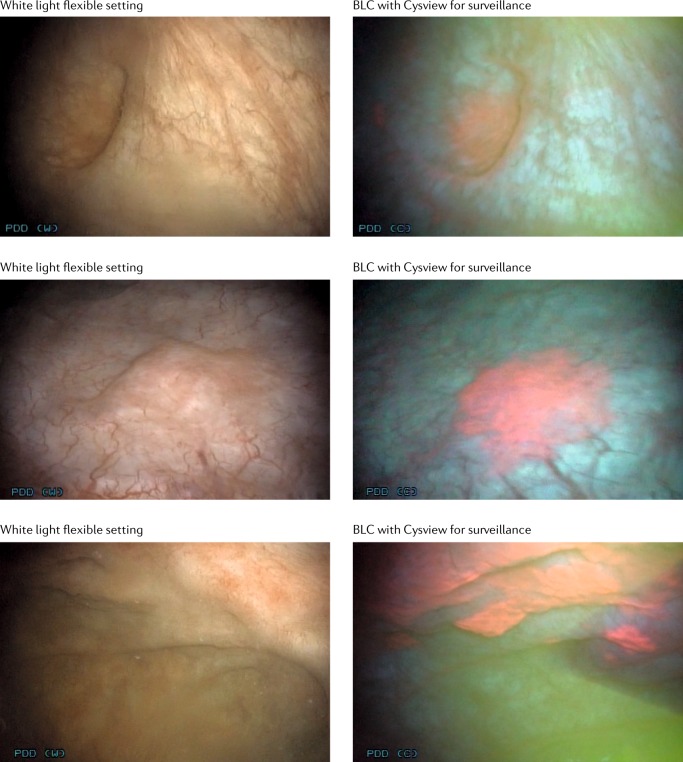
Detection of non-muscle-invasive bladder cancer with flexible white light and blue light cystoscopy with Cysview. Blue light images depict the same area as white light cystoscopy but demonstrate pink lesions in areas of malignancy. Images are previously unpublished from the phase III multicentre trial involving Photocure^10^. BLC, blue light cystoscopy.

BLC is an FDA-approved photodynamic diagnostic technique that serves as an adjunct to WLC to improve visualization and treatment of bladder cancer^9^. Intravesical instillation of the haem precursor hexaminolevulinate (HAL; which is known as Hexvix in Europe and Cysview in the USA) before cystoscopy results in preferential accumulation of protoporphyrin IX and other photoactive porphyrins in neoplastic tissue, which fluoresces red when exposed to blue light^10,11^ (Fig. 1). Up until the approval of BLFC for surveillance in the USA in 2018 (ref.^12^), BLC was able to be used only in the operating room. BLC has several benefits, including improved detection of CIS and papillary tumours as well as reduction in recurrence and progression compared with WLC. Multiple prospective studies have compared BLC and WLC for managing patients with NMIBC and demonstrate significantly (*P* < 0.05) improved detection rates with BLC, in particular for CIS, Ta and high-grade tumours^8,13–15^. A meta-analysis by Burger et al.^8^ found that 24.9% of patients had at least one Ta or T1 tumour detected only by BLC and 26.7% of patients had at least one CIS lesion detected only by BLC (*P* < 0.001). This demonstration of improved detection served as an impetus to incorporate BLC technology into outpatient surveillance.

The goal of improved detection in the operating room is to improve the quality of TURBT, in turn reducing the risk of recurrence. Grossman et al.^16^ published long-term follow-up data from 551 participants enrolled in a prospective, randomized study comparing BLC with WLC for Ta or T1 urothelial bladder cancer. After a median follow-up duration of 53.0 months in the white light group and 55.1 months in the fluorescence group, the recurrence-free rate was 31.8% and 38% in WLC and BLC arms, respectively (*P* = 0.14). Median time to recurrence was 9.4 months for patients who received WLC and 16.4 months for those who underwent BLC (*P* = 0.04). Preliminary data on the effect of BLC on recurrence were published in 2012 (ref.^16^), and many subsequent studies investigating the effect of BLC on recurrence and progression in NMIBC have since been published (Table 1). Early recurrence rates at first follow-up cystoscopy, 2-year recurrence rates and 3-year recurrence rates were all lower with BLC than WLC^17–19^. The lower recurrence rates associated with BLC than WLC alone have been demonstrated for multiple clinical scenarios, including single tumours, multiple tumours, tumours <3 cm and tumours >3 cm (ref.^17^). In a meta-analysis of six studies including 831 patients, recurrence rates up to 12 months were significantly lower overall with BLC, 34.5% versus 45.4% (*P* = 0.006; risk ratio (RR) 0.761 (95% CI 0.627–0.924)), than with WLC and were reduced in patients with T1 or CIS tumours (*P* = 0.052; RR 0.696 (95% CI 0.482–1.003)), Ta tumours (*P* = 0.040; RR 0.804 (95% CI 0.653–0.991)) and in high-risk (*P* = 0.050) and low-risk (*P* = 0.029) subgroups^8^. Similar findings of decreased recurrence have also been reported in other studies^8,17–19^ (Table 1).

**Table 1:** Studies comparing recurrence rates using BLC and WLC

Factor	Burger et al.^8^	Geavlete et al.^18^		Mariappan et al.^17^	Gallagher et al.^19^	
Number of patients included	2,212	362	362	808	808	808
Time to follow-up appointment	1 year	3 months	1 year	First follow-up cystoscopy	1 year	3 years
Recurrence rate, BLC	34.5%	7.2%	31.2%	13.6%	21.5%	39.0%
Recurrence rate, WLC	45.4%	15.8%	45.6%	30.9%	38.9%	53.3%
*P* value	0.006	0.003	0.001	<0.001	<0.001	0.02

BLC, blue light cystoscopy; WLC, white light cystoscopy.

Interest in whether BLC will also reduce progression to muscle-invasive disease is considerable. Progression is a major concern in patients with high-risk NMIBC as it can result in additional morbidity and mortality^2^. Gakis et al.^20^ performed a systematic literature review to evaluate the effect of BLC during TURBT on progression compared with WLC and found five studies (four randomized and one retrospective). In a total of 1,301 patients, 644 underwent BLC-based and 657 WLC-based TURBT^20^. Progression was reported in 44 of 644 patients (6.8%) who received BLC and 70 of 657 patients (10.7%) who underwent WLC (median OR 1.64, 95% CI 1.10–2.45 for BLC versus WLC; *P* = 0.01).

Kamat et al.^21^ used a new definition of disease progression in NMIBC proposed by the International Bladder Cancer Group. Previously, progression was considered only in patients who progressed from NMIBC to muscle-invasive bladder cancer (stage T2 or greater). The new definition would also include change in grade from low to high grade. A re-analysis of a controlled phase III study in patients with NMIBC who received BLC (255 patients) or WLC cystoscopy (261 patients) using the new definition showed that 31 patients (12.2%) who received BLC versus 46 (17.6%) who received WLC (*P* = 0.085) experienced disease progression. Efforts to determine the effect of BLC on progression are ongoing^22^.

## Experience with BLFC

### The Nordic perspective

The variation in incidence rates of bladder cancer between geographical regions of the world is tenfold, with the highest rates in Europe and North America compared with lower incidence in Central and South America and Asia^23^. The national cancer registries in the Nordic countries, started in the 1950s, are compulsory by law and were the first of their kind in the world. According to the combined Nordic cancer registries — Nordcan — the age-standardized incidence of bladder cancer more than doubled for both genders until the 1990s but then stabilized. However, the overall incidence has continued to increase but at a somewhat lower rate than before the 1990s^24^.

Hexvix was initially approved in Sweden in 2004 and was later approved in each country of Europe, with Switzerland last in November 2007 (ref.^25^), and as Cysview in the USA in 2010 (ref.^26^). The benefits of using this diagnostic method in the operating room led to its recommended use in the AUA and EAU guidelines^2,4,27–29^. European approval was not limited to certain equipment, but initial use was primarily for rigid cystoscopies in the operating room, with only a few reports of use with flexible scopes^27,28^. The ongoing technical development and miniaturization of equipment, such as fibre optical (and later digital) flexible cystoscopes with outstanding image quality combined with the possibilities of a working channel, have initiated an interest in the use of this method in the outpatient setting.

Zare et al.^30^ published a multicentre prospective observational assessment of BLFC for surveillance and fulguration that included 69 patients with a mean age of 70 years (range 33–89 years) and a mean duration since NMIBC diagnosis of 8 years. Most patients had high-grade cancer at initial diagnosis (52 of 69) and were at high risk of recurrence (48 of 69). Overall, two patients per hour could be assessed using outpatient BLFC. Preparation and instillation of HAL took <10 minutes per patient, and patients had an additional waiting time of 45–60 minutes while the HAL solution was retained in the bladder before examination. In total, 11 patients had histologically confirmed tumours identified using both WLFC and BLFC. An additional three patients had tumours identified only by BLFC — two with Ta tumours and one with CIS. Of the 14 patients in total with confirmed tumours, 11 could be managed concomitantly with fulguration, whereas 3 were referred to the operating room. No adverse events attributable to BLFC were reported^30^.

**Fig. 2:**
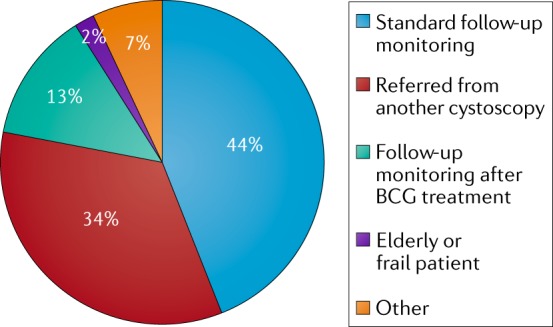
Indications for BLFC. Initial Nordic experience of indications for using blue light flexible cystoscopy (BLFC). Overall, the most frequent indication for BLFC was for standard follow-up monitoring (44%), followed by referral from another cystoscopy (34%) and follow-up monitoring after BCG treatment (13%). Being frail or an elderly individual was the least frequent indication (2%)^31^.

**Fig. 3:**
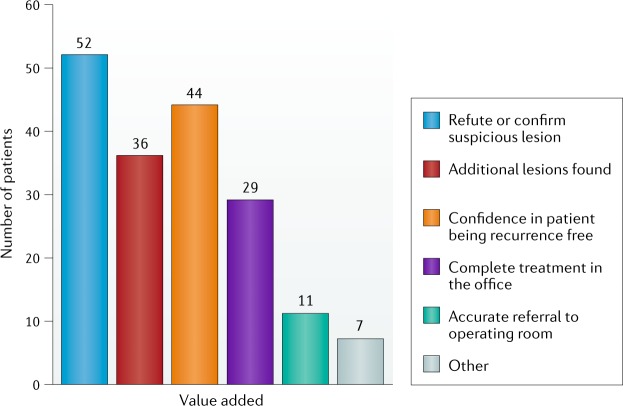
Perceived value of BLFC by clinicians. Added value was reported in 85% of procedures. One or more additional value could be listed per procedure. Blue light flexible cystoscopy (BLFC) added value for refuting or confirming suspicious lesions in 52 patients. In 36 patients, additional lesions were found, and in 44 patients, BLFC enabled the clinician to be confident that the disease had not recurred. For 29 patients, the procedure was able to be completed in an office setting, and BLFC enabled accurate referral to the operating room in 11 patients^31^.

A Nordic registry has been established that collects data on the potential benefit of using BLFC with HAL in patients with suspicion of NMIBC or in routine follow-up monitoring after therapy for confirmed NMIBC^31^. The aim of this registry is to provide improved understanding of which patient populations might benefit from BLFC, the effect on treatment decisions and outcomes, the feasibility of procedures, tolerability and patient preference. In the first analysis of the registry data, 178 visits by 136 patients were reported^31^. The average age was 73 years and 23% were women. Of the patients included, their previous stages of bladder cancer were Ta (60% of patients), T1 (20%) and Tis (20%). Overall, 49% of the patients had high-grade disease. The most common indications were referral after suspicious WLC, follow-up monitoring after BCG and standard follow-up monitoring (Fig. 2). In total, 83% of patients who underwent procedures received cystoscopy and fulguration in the office, and most patients preferred treatment in the outpatient setting to standard TURBT. Physicians reported an added value of BLFC in 85% of patients (Fig. 3).

The key advantages noted with BLFC were additional lesions found, confirmation or refutation of suspicious lesions, confidence in patients being free of recurrence and complete ablation in the office^26^. In summary, this first analysis showed that BLFC can be easily introduced into the clinical routine for patients with NMIBC, preventing unnecessary referral to surgery when small lesions can be treated in the office.

This European experience demonstrates that introduction of BLFC in the clinic can be successful without considerable logistical issues and is beneficial, especially when coupled with tumour ablation procedures.

### The US experience

#### Phase III study of BLFC for surveillance

**Fig. 4:**
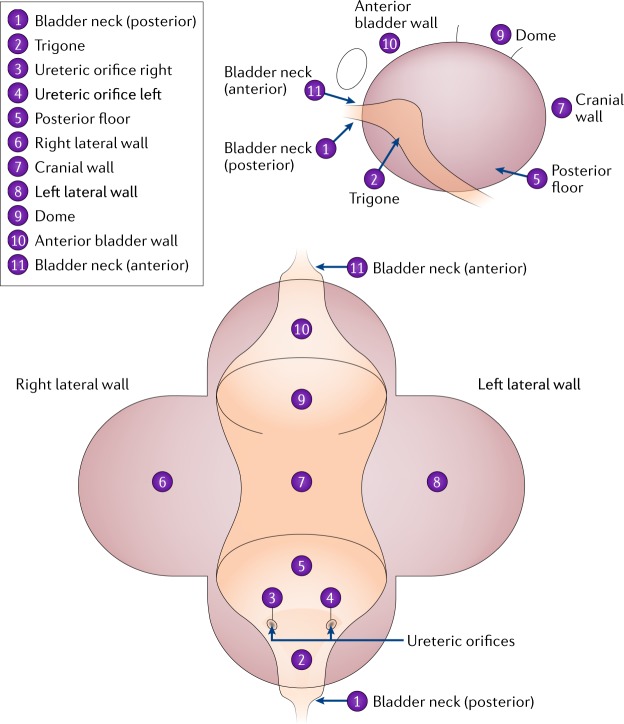
The bladder map used in a phase III multicentre study^10^ to graph location of suspicious lesions. An example of a bladder map. This bladder map was used in a phase III multicentre study^10^ to graph location of suspicious lesions.

In 2018, the benefit of BLFC was evaluated in the USA in a prospective, multicentre study^10^. This trial was an open-label, comparative, within-patient, controlled phase III study performed at 17 centres^10^. The study enrolled 304 patients, including 202 who had previously had high-grade tumours. Those patients who were eligible required a history of multiple, recurrent or high-grade bladder tumours. Patients were excluded if they had received BCG immunotherapy or intravesical chemotherapy in the previous 6 weeks. This cut-off value was used to reflect guideline-directed clinical practice as surveillance cystoscopy is often performed 6 weeks after last instillation. At the first office-based surveillance visit, all patients underwent cytology (but information from this test was not used in decision-making) and then received an instillation of HAL. Patients then underwent flexible cystoscopy in an office setting under local intraurethral anaesthesia^10^. Initial evaluation was with WLFC, and number, size and appearance of all suspected malignant lesions were documented using a bladder map (Fig. 4). Following WLFC, patients were randomized either to undergo BLFC or not to undergo BLFC, and suspicious lesions were again recorded. The randomization was included to make sure the initial WLFC was performed optimally as the urologist would not know if a BLFC would be performed subsequently. Re-examining the bladder with white light was not allowed in order to minimize bias. Patients with suspicious findings under either method were referred to the operating room according to study protocol and underwent evaluation and resection WLC or BLC depending on which procedure they had previously received. A consensus panel of pathologists, blinded to the method, determined the final pathology^10^.

The primary efficacy end point was the proportion of patients with histologically confirmed malignancy that was detected only by BLFC and not by WLFC in the surveillance setting. Secondary efficacy end points regarded the operative resection, which included the proportion of patients with CIS lesions or additional tumours identified with BLFC but not seen with WLFC (Fig. 1). The primary safety end point was the proportion of patients with adverse events following surveillance^10^.

The tumour stage at previous TURBT was CIS in 100 patients (33%) and T1 in 52 patients (17%). Previous recurrence of 1–4 tumours was experienced by 184 patients (61%, mean 1.7 ± 2.03). Intravesical therapy with BCG or chemotherapy within the previous 90 days had been received by 202 patients (66%). In total, 103 patients were taken to the operating room owing to a suspicious lesion identified during surveillance, of whom 63 (41%) had confirmed bladder cancer. In evaluation of the primary efficacy end point, 13 patients (20.6%, 95% CI 11.5–32.7) had malignant recurrences detected only with BLFC (*P* < 0.0001), and 5 of these were confirmed as CIS. WLFC detected only one malignant recurrence (1.6%) that was not seen with BLFC. The false-positive rate was 9.1% in both the WLFC and BLFC arms. This rate is consistent with previous BLC meta-analysis^32^ and highlights that although the majority (66%) of patients had undergone either intravesical immunotherapy or chemotherapy within 6 weeks to 90 days, the resulting inflammation did not result in an increased false-positive rate compared with the previous recommendation to wait 90 days before using BLC. Of the 63 patients with recurrent tumours, 7 had suspicious cytology. Of the 103 patients who went to the operating room, 13 had suspicious cytology. When considering only patients with CIS (*n* = 26), only 7 patients had suspicious cytology. In fact, tumours in 9 of the 26 patients were found using blue light (combining those found using BLFC in the clinic and BLC in the operating room) alone, and, of these patients, 0 had positive cytology, 1 had suspicious cytology results, 4 had atypical results, 3 had negative cytology and 1 had missing results. These findings highlight the need for enhanced cystoscopy, as relying on cytology to detect CIS missed by WLC is likely to be inadequate for finding most cancers^33^. The additional detection of tumours with BLFC in this study is consistent with analyses of multiple phase III studies with detection of CIS by BLC alone, which identifies 20–40% of CIS tumours, resulting in a significantly improved detection rate of 0.87 for BLC versus 0.75 for WLC (*P = *0.006)^13,34^. In the current study, combined BLC and BLFC also detected additional malignant lesions that were missed with WLC and WLFC in 29 of the 63 patients (46%, 95% CI 33.4–59.1), which is consistent with previous phase III studies^14,15^. Currently, no follow-up data are available for analysis; thus, rates of recurrence or progression are unknown.

## Patient-reported outcomes

Evaluating new technologies requires assessment of efficacy (such as cancer detection for BLC) and the effect of a technology on patient experience (for example, their feelings of pain and/or anxiety). BLFC requires additional catheterization for instillation of HAL and a subsequent waiting period for HAL uptake into the urothelium^9,11^; in addition, pain and anxiety are potentially associated with the procedure. The implications of findings (positive or negative) on patients’ anxiety and views on whether it was worthwhile also require consideration.

The outcomes of patients enrolled in the BLFC for surveillance phase III clinical trial^10^ were assessed at baseline, immediately following surveillance cystoscopy and, for those referred to the operating room, after they had received the pathology findings and were aware of their diagnosis^35^. The Patient-Reported Outcomes Measurement Information System (PROMIS) Anxiety 4a form was used to measure anxiety and the PROMIS Pain Intensity 1a form was used to rank pain on a scale from 0 (no pain) to 10 (worst pain). Following each procedure, patients were queried regarding their willingness to pay and the value of the test using the Was it Worth It (WIWI)? questionnaire.

Pain levels were low throughout the study. Mean pain scores were low for patients at baseline (0.6 (s.d. 1.51)), post-surveillance (1.1 (s.d. 1.92)) and postoperating room (1.4 (s.d. 2.13))^35^. Anxiety decreased uniformly after BLFC (Δ = –2.6), with no differences on the basis of gender, BLFC results (positive or negative) or test performance (false positive or true positive). Most patients found BLFC “worthwhile”, “would do it again” and “would recommend to others”, with no differences on the basis of BLFC result or test performance. Furthermore, most patients (76%) were willing to pay themselves, with >50% willing to pay $100 or more for BLFC with HAL^35^.

Members of the consensus panel acknowledged several limitations and research gaps, including the absence of a WLFC comparator and the value of additional questionnaires such as health-related quality of life. The panel discussed the merit of future patient-reported outcome comparison between WLFC and BLFC for surveillance as well as the inclusion of patient-reported outcomes in longitudinal registry studies. The consensus panel noted the need to evaluate the cost-effectiveness of the technology for patients in different risk groups.

## Safety

An important aim of the phase III study evaluating BLFC^10^ was assessment of its safety regarding repeated administration of HAL and the use of BLFC after intravesical BCG. Patients included in the trial were specifically questioned about adverse events after completing the surveillance cystoscopy, during the operating room exam and 1 week after completing each of these procedures. Each individual investigator determined whether the recorded adverse events were probably caused by HAL and whether they should be deemed serious. Before study entry, 82.2% of patients had received previous intravesical BCG and 37.5% had received previous intravesical chemotherapy. Of these patients, 66.7% received their last dose of intravesical therapy between 6 and 12 weeks before the surveillance cystoscopy visit^10^.

At the surveillance visit, 12 adverse events (in 11 patients) were recorded out of a total of 304 patients, a rate of 3.6%. In the operating room, 13 adverse events were recorded (in 8 patients) out of a total of 103 patients, a rate of 7.8%. In the judgement of the investigator, HAL-related adverse events were experienced by six patients during surveillance (2.0%) and by three patients following the operating room exam (2.9%). These adverse events included dysuria or urethral pain, bladder discomfort, erythema and pruritus following surveillance as well as procedural pain and contact dermatitis following repeat use of HAL in the operating room visit. Other than the 103 (33.8%) patients who received two doses of HAL during the study period per protocol, an additional 122 (40.1%) patients received at least two lifetime doses of HAL, including 90 (29.6%) patients who received three or more doses of HAL^10^.

Previous exposure to HAL did not affect the likelihood of adverse events compared with patients with no previous exposure to HAL. The timing of repeat use of HAL also had no effect on the adverse event profile, including in the 92 patients who received at least two instillations within a period of 45 days. Repeat instillation was, therefore, deemed to be safe by the FDA, and on the basis of these results the FDA lifted the restriction on single use in 2018 (ref.^12^). Furthermore, the rate of adverse events during the surveillance cystoscopy visit did not seem to be related to previous administration or timing of intravesical therapy, including those patients who received their last dose of the drug within 60 days of HAL administration^10^. The consensus panel agreed that BLFC was safe and could be performed repeatedly and within 60 days of instillation of BCG.

## Consensus panel conclusions

### BLFC for surveillance: best practice

Several factors need consideration for implementing BLFC for surveillance. The main goal of surveillance of bladder cancer is to determine whether a patient has recurrence or progression. Thus, all patients could benefit from enhanced cystoscopy either to reassure the patient and provider that no cancers were missed or to identify suspicious areas that need biopsy or, at a minimum, closer attention. However, BLFC requires an extra step of instillation, increased time (the patient needs to arrive an hour earlier than for WLFC) and increased resources and initial expense. Thus, early in the adoption of BLFC, selecting patients who are most suitable for the procedure will probably be beneficial. Accumulating evidence might further clarify the role of BLFC for different disease-risk and specific scenarios; however, the goal of this consensus panel was to make recommendations regarding likely benefit of use of BLFC (Table 2).

**Table 2:** Consensus recommendations for best practice in the use of BLFC for surveillance

Recommendation number	Factor	Consensus panel recommendation
1	Likelihood of recurrence	Strong recommendation for value of BLFC at initial 3-month cystoscopy for patients at high risk (100% of the panel) or intermediate risk (71% of the panel) of recurrence according to AUA guidelines
2	Frequency of use	Most panellists (94%) recommended BLFC at 3 and 6 months and then every 6 months for patients at high risk of recurrence in the first 2 years
3	Specific clinical scenario: residual disease	Most panellists (76%) recommended use before intravesical therapy if residual disease after TURBT is a concern
4	Specific clinical scenario: biopsy and/or fulguration	Most panellists (76%) recommended for use at time of office fulguration and/or biopsy for low-grade tumours
5	Specific clinical scenario: positive cytology and normal WLC and equivocal lesions with negative WLFC	Might have a role in evaluating patients with a positive cytology and normal WLC (88% of the panel) or equivocal lesions on WLFC with negative cytology (63% of the panel)

AUA, American Urological Association; BLFC, blue light flexible cystoscopy; TURBT, transurethral resection of bladder tumour; WLC, white light cystoscopy; WLFC, white light flexible cystoscopy.

#### Panel recommendations: likelihood of recurrence

The first factor considered by the expert panel was likelihood of recurrence, which is highest at the 3-month surveillance visit^2,36^. The entire panel agreed that patients at high risk of recurrence would benefit from BLFC for surveillance at 3 months. Similarly, 71% of panel members agreed that patients at intermediate risk of recurrence could benefit as well, as they already demonstrated a propensity for either multiple tumours or multiple recurrences. However, 88% of panellists did not think BLFC for surveillance was worthwhile in patients at low risk of recurrence. The rationale for this conclusion was that these patients are unlikely to progress even if a small low-grade tumour is missed by WLC (Table 2).

#### Panel recommendations: frequency of surveillance

The second factor focused on frequency of use during surveillance in the first 2 years. In patients at low risk of recurrence, no clear consensus was agreed upon for frequency of use, but 94% would not use BLFC in these patients. In intermediate-risk disease, divergence of opinions among panellists on frequency of use was evident, with 18% supporting 6-month intervals and 29% supporting annual intervals. Furthermore, 35% thought frequency of use depended on the findings at the 3-month cystoscopy evaluation, and 18% did not think they would use BLFC routinely for recurrent low-grade tumours. For patients at high risk of recurrence, 94% agreed that BLFC would be of value at 3 months, 6 months and every 6 months for 2 years given the high risk level. Approximately 53% of panel members would use BLFC every 3 months for the first year. Determining BCG unresponsiveness, especially in patients with CIS, was felt to be an important end point for which BLFC would be specifically useful during surveillance (Table 2).

#### Panel recommendations: specific clinical scenarios

Several specific clinical scenarios in which BLFC was thought to have utility were identified (Table 2). In patients in whom residual disease is a concern, such as those who had undergone TURBT performed elsewhere or without BLC, 76% of the panel thought that an office BLFC would be of benefit before initiating intravesical therapy in patients at intermediate risk or high risk of recurrence. Similarly, 76% of the panel agreed that, in patients undergoing office biopsy and fulguration, BLFC would be useful to ensure all lesions were identified. This procedure would be particularly useful in patients with small, multiple or recurrent low-grade papillary tumours.

For patients with positive cytology and normal WLC, the AUA guidelines state that “In a patient with a history of NMIBC with normal cystoscopy and positive cytology, a clinician should consider prostatic urethral biopsies and upper tract imaging, as well as enhanced cystoscopic techniques (BLC, when available), ureteroscopy, or random bladder biopsies (Expert Opinion)”^2^. Overall, 88% of the panel agreed that BLFC could be helpful in patients with a positive cytology and normal WLC. BLFC has improved detection of CIS^8,10^; thus, knowing whether enhancing lesions are present before operative intervention could be useful. Obtaining tissue in the clinic setting could be possible. Furthermore, in patients without suspicious lesions, upper tract imaging or selective upper tract cytology might be necessary. In addition, in patients who are poor surgical candidates, BLFC might help to determine the diagnosis without needing to go to the operating room.

Another scenario in which BLFC might be of benefit is in patients with equivocal lesions (erythema) during WLFC with negative cytology, with 63% of the panel agreeing with this use. These patients might have cancer, but biopsy in the operating room can be associated with morbidities. Using a short-interval (4–6 weeks) BLFC might help to identify which of these lesions is most likely to be cancerous as opposed to false-positive WLC lesions.

### Practicalities of implementation

Incorporating new technology into the clinic requires a stepwise approach. The initial consideration is acquisition of equipment: to start using BLFC, the Karl Storz D-Light C PDD Flexible cystoscopy system needs to be acquired as it is the only approved cystoscope at this time in the USA. For the USA, Cysview kits (Photocure) need to be ordered. Furthermore, office assistants and staff need to be educated regarding assembly and instillation.

Once equipment has been obtained, the next step is to identify the appropriate patients for the procedure. As mentioned above, many patients could benefit from BLFC. The ideal situation for long-term use, in the opinion of the panel, is to identify patients when reviewing pathology after a TURBT. In appropriate patients, the next office cystoscopy can be scheduled as WLFC or BLFC and then the patient can be informed as to whether they need to arrive early and office staff can have the appropriate scope and equipment scheduled. In the interim, the upcoming clinic schedules can be reviewed to identify appropriate patients. In addition, for patients undergoing office cystoscopy for whom small tumours are identified and office cystoscopy and fulguration and/or biopsy are planned, then BLFC can be scheduled. Similarly, for other specific scenarios noted above, such as equivocal WLFC lesions for which BLFC might offer a benefit, the procedure can be scheduled.

Other logistical considerations need to be taken into account as the instillation of HAL requires a 1-hour dwell time; contacting patients to remind them to arrive at least 60 minutes before the procedure to allow time for instillation is important to ensure the procedure schedule is not disrupted. Other considerations include the sterilization method, as Karl Storz and Olympus scopes require different sterilization methods. The number of scopes and towers required, which could limit the number of procedures that can be performed at each clinic, needs consideration. In addition, all the providers who might decide to perform BLFC needs to be determined so that conflict between schedules does not occur. Detailed instructions for use and integration into electronic medical record technologies can be helpful when identifying patients and scheduling blue light procedures.

In addition, Cysview HAL instillation has a specific Healthcare Common Procedure Coding System code (A9589) for use in the clinic and physician office or clinic setting of care for BLFC for surveillance^37^. Other codes are also used, including a BLC complexity adjustment code (C9738) for certain BLC procedures, which will result in incremental reimbursement^38^.

## Future directions

Several areas could benefit from further research regarding the use of BLFC. Most of the patients in the prospective phase III study^10^ were at a high risk of recurrence; thus, the role in patients at low risk is unclear. Furthermore, the mandatory requirement of the study to perform biopsies on all abnormal lesions probably skewed the false-positive rate as some clinicians would choose to monitor some lesions or await cytology before going to the operating room to perform a biopsy. Moreover, office biopsy samples could be used to evaluate indeterminate lesions in some patients, which would reduce the morbidity caused by taking patients to the operating room.

A blue light registry exists and currently includes >1,250 patients and will start including patients who have received BLFC, which will help answer some of these questions related to the utility of BLFC in the outpatient setting. The first paper from the registry was published in 2018 and showed that BLC increases detection rates of CIS and papillary lesions over WLC alone and can change management in 14% of patients. This registry can help answer unanswered questions about the efficacy of BLFC in low-risk and intermediate-risk disease and the effect on long-term outcomes^22,32^. Furthermore, the cost-effectiveness of introducing new technologies is an important issue, and BLFC needs to be evaluated as bladder cancer is one of the most expensive diseases to manage owing to the need for lifelong surveillance. The advantages of early detection and the effect on recurrence rates and treatment selection will need to be balanced against the increase in costs and time associated with use. Lastly, the conceptual design and therapeutic efficacy of photodynamic therapy has been evaluated in NMIBC^39^. Photodynamic therapy can be defined simply as a form of treatment that uses a photosensitizing agent that concentrates selectively in malignant cells and, following exposure to ultraviolet light, can destroy the specific cell types in which it localizes^40^. By administering intravesical Cysview (HAL), protoporphyrin IX specifically accumulates in excess in urothelial cancer cells (particularly CIS and papillary carcinoma). The excess accumulation of protoporphyrin IX molecules that occurs means they can be excited by specific wavelengths of light. A prospective study of 17 patients used HAL (Hexvix) with 50 ml of a 16 mM (4 patients) or 8 mM HAL (13 patients) solution instilled intravesically^40^. Bladder wall irradiation was performed using an incoherent white light source (T-Light; Karl Storz). The light source comprised a 500 W short arc xenon bulb emitting light in the visible spectrum from 380 to 700 nm. A total of 2–6 W could be transmitted through a single 1.5 mm diameter quartz fibre. The first 14 patients treated had a target light dose of 100 J/cm^2^ and received three treatments with HAL photodynamic therapy 6 weeks apart. Another three patients had a target light dose of 25 J/cm^2^ at the first photodynamic therapy session, 50 J/cm^2^ at the second and 100 J/cm^2^ at the third. Preliminary assessment of efficacy showed that of the 17 patients included, 9 (52.9%, 95% CI 27.8–77.0) were tumour free at 6 months, 4 (23.5%, 95% CI 6.8–49.9) were tumour free at 9 months and 2 (11.8%, 95% CI 1.5–36.4) were tumour free after 21 months.

Well-designed clinical trials to test the utility of Cysview as a photodynamic therapy are warranted, and this treatment is a novel precision-based approach that is less invasive in the management of NMIBC than current intravesical treatments.

## Conclusions

A phase III, prospective, multicentre study^10^ found that BLFC improves detection of bladder cancer compared with WLFC for surveillance. Toxic effects were minimal, and most patients found it to be worthwhile and would recommend BLFC to others. Anxiety decreased after surveillance with BLFC. Consensus was reached regarding the benefit of using BLFC at the initial 3-month cystoscopy for patients at high risk or intermediate risk of recurrence according to AUA guidelines. Other recommendations from the expert panel were to use BLFC during surveillance of patients at high risk of recurrence at 3 and 6 months and then every 6 months in the first 2 years. BLFC is also recommended in patients before intravesical therapy if residual disease after TURBT is a concern, at the time of office fulguration and/or biopsy for low-grade tumours and in evaluating patients with a positive cytology or equivocal lesions on WLC. Further research will clarify the role of BLFC in patients at low or intermediate risk of recurrence, as a tool during office biopsy and fulguration and in photodynamic therapy. A prospective registry will provide additional data on outcomes and cost-effectiveness.
